# *Mayaweckelia troglomorpha*, a new subterranean amphipod species from Yucatán state, México (Amphipoda, Hadziidae)

**DOI:** 10.3897/zookeys.735.21164

**Published:** 2018-02-06

**Authors:** Dorottya Angyal, Efraín Chávez Solís, Benjamín Magaña, Gergely Balázs, Nuno Simoes

**Affiliations:** 1 Department of Zoology, Hungarian Natural History Museum, Baross u. 13, 1088 Budapest, Hungary; 2 Laboratory of Molecular Taxonomy, Hungarian Natural History Museum, Ludovika tér 2-6, 1083 Budapest, Hungary; 3 Multidisciplinary Teaching and Research Unit of Sisal, Faculty of Sciences, Academic Unit of Yucatán, National Autonomous University of México, (UMDI-Sisal, FC, UNAM), Puerto de abrigo S/N, C.P. 97356, Sisal, Yucatán, México; 4 Biological Sciences Postgraduate Program, National Autonomous University of México, Avenida Universidad 3000, Copilco-Universidad, México City 04510, México; 5 Department of Systematic Zoology and Ecology, Eötvös Loránd University, Pázmány Péter sétány 1/C, 1117 Budapest, Hungary; 6 National Coastal Resilience Laboratory (LANRESC), Puerto de abrigo S/N, C.P. 97356, Sisal, Yucatán, México; 7 International Chair for Ocean and Coastal Studies, Harte Research Institute, Texas A&M at Corpus Christi, Texas, USA

**Keywords:** cenote, description, endemic, Hadziidae, mitochondrial marker, morphology, SEM, sinkhole, subterranean

## Abstract

A detailed description of a new stygobiont species of the amphipod family Hadziidae, *Mayaweckelia troglomorpha* Angyal, **sp. n.** is given, based on material collected in four cenotes of Yucatán federal state, México. Morphology was studied under light microscopy and with scanning electron microscopy. Morphological description is complemented with mitochondrial cytochrome c oxidase subunit I (COI) sequences as barcodes, with affinities to the related taxa and with notes on the species’ ecology. Using COI Bayesian inference and genetic distance analyses, we show that the closest relative of the new species is *M. cenoticola*, forming a monophyletic group referring to the genus *Mayaweckelia*. Based on the available sequences, we also revealed that *Mayaweckelia* and *Tuluweckelia* are sister genera, standing close to the third Yucatán subterranean genus, *Bahadzia*. The data gathered on the habitat, distribution, abundance, and ecology will contribute to the conservation planning for *M. troglomorpha* Angyal, **sp. n.**

## Introduction

To date, eleven species of amphipods have been recorded from subterranean habitats of the Yucatán Peninsula, belonging to five families (Ampithoidae, Hadziidae, Hyalidae, Hyalellidae, and Melitidae) (e.g., Holsinger 1977, 1990, 1992, Alvarez and Iliffe 2008, Marrón-Becerra et al. 2014, Ortiz and Winifield 2015, Trujillo-Pisanty et al. 2010). Among them, six species are classified as stygobionts; they bind solely to aquatic subterranean habitats, exhibiting various degrees of morphological, physiological and behavioural adaptations to life in the hypogean environment (Notenboom 1991). These six species are endemic to the Yucatán Peninsula and inhabit mostly ’cenotes’ (also referred as sinkholes), well-like water-filled karst features, which are formed by the collapse of limestone bedrock and are usually connected with extended submerged cave passages (e.g., Reddell 1981, Alvarez et al. 2015). Like other karst aquifers, the Yucatán cenotes are particularly vulnerable to contamination especially from tourist activities and infrastructure, pollution from growing human settlements, industrial and agricultural activities (Escolero et al. 2002, Bauer-Gottwein et al. 2011).

Cenotes of the Yucatán Peninsula are considered anchialine environments; they are filled with fresh and saltwater, separated by a halocline layer (Bauer-Gottwein et al. 2011). Contrary to the cenotes found on the Caribbean coast of the peninsula, those in Yucatán federal state (which is located on the north part of the peninsula, bordered by Campeche federal state to the southwest and Quintana Roo federal state to the east, with the Gulf of México on its north coast) are mainly inland, far from the coastline and therefore are filled with freshwater only. The saline intrusion can only be detected in a few rather deep cenotes, like Sabak-Ha (20.579974°N, 89.588353°W, halocline at 62 m, own data) and Ultimo Suspiro (21.403485°N, 88.568434°W halocline at 51 m, own data), or in few cenotes, which are located near the northern coast of the peninsula, like Cervera, in which the halocline occurs at about 25 m depth (Alvarez et al. 2005).

The hadziid *Tuluweckelia cernua* Holsinger, 1990, *Bahadzia bozanici* Holsinger, 1992 and *Bahadzia setodactylus* Holsinger, 1992 and the hyalellid amphipod *Hyalella cenotensis* Marrón-Becerra, Hermoso-Salazar & Solís-Weiss, 2014 have been described from caves and cenotes of Quintana Roo state near the Caribbean (eastern) coast of the peninsula (Holsinger 1990, 1992, Marrón-Becerra et al. 2014), while *Mayaweckelia yucatanensis* Holsinger, 1977 (Hadziidae) is reported from a cave pool in Campeche state (Holsinger 1977). The only stygobiont amphipod that has been described from Yucatán state is *Mayaweckelia cenoticola* Holsinger, 1977.

In his genus description where Holsinger (1977) described the type species *M. yucatanensis* and *M. cenoticola*, he highlighted the differences from the two known related subterranean hadziid genera, *Mexiweckelia* Holsinger & Minckley, 1971 and *Hadzia* S. Karaman, 1932. The new genus differs in some important characters, such as the three-articulated accessory flagellum of the first antenna, the absence of robust setae on the inner margin of the maxilliped outer plate, the presence of ventrally produced lobe of gnathopod I merus and the absence of dorsal robust setae on urosomites I and II. Other diagnostic characters of *Mayaweckelia* are the absence of the mandibular palp, the completely separated telson halves, and the outer ramus of the third uropod with one article (Holsinger 1977). In his paper written about the description of the genus *Tuluweckelia* and the type species *T. cernua*, Holsinger (1990) also gave a second, complementary description of *M. cenoticola*, where he presented some characters that were found since the original description as: the presence of aesthetascs on flagellar articles ten-twelve on the first antenna, pereopod VI up to 15% longer than pereopod VII and the presence of a row of fine setae on the distal half of upper margin of pereopods V-VII dactyli.

This study results from a long-term research project using cave diving techniques, initiated in May 2016 to contribute to the understanding of Crustacea diversity and distribution in the cenote ecosystems of Yucatán federal state (Angyal et al., in preparation). As part of the project findings, the description of a new species of *Mayaweckelia* is presented herein, using several sources of data that increase the robustness of taxonomic conclusions (Padial et al. 2010). Cytochrome c oxidase subunit I (COI) sequences are provided as barcodes, as well as the first comparative scanning electron micrographs (SEM) of *Mayaweckelia*. Moreover, phylogenetic relationships based on mitochondrial sequences of the collected *Mayaweckelia* and *Tuluweckelia* samples are presented, including publicly available hadziid sequences. Field observations that may contribute to the species’ conservation and ecological comprehension are summarised.

## Materials and methods

### Sampling sites and sampling

**Figure 1.**
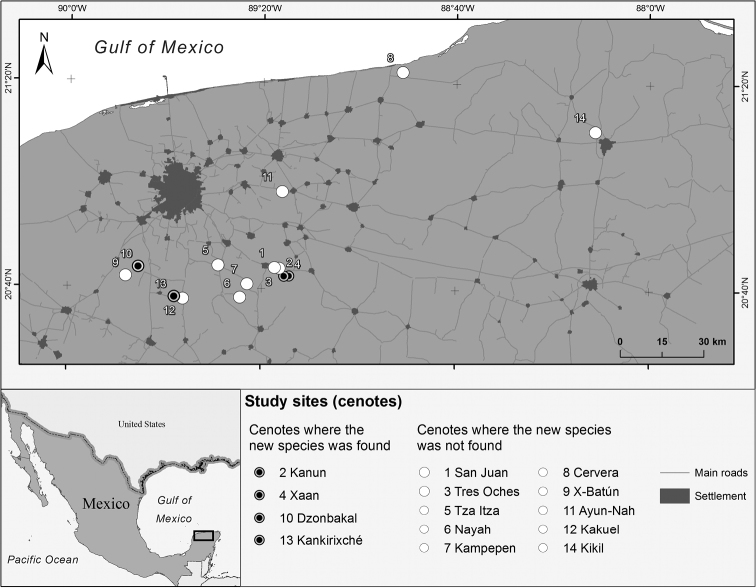
Location of the studied area, showing the four cenotes where the new species was collected (Yucatán federal state, México).

Fourteen cenotes were studied between May and July 2016 in seven municipalities of Yucatán federal state (Yucatán Peninsula, México) in order to characterize their Crustacea fauna (Angyal et al., in preparation). Most of these cenotes are situated near Mérida city and are part of the ’Ring of cenotes’, which is a fracture zone that marks the outline of the Chicxulub asteroid impact crater with a high density of sinkholes (Gonzales-Herrera et al. 2002, Bauer-Gottwein et al. 2011). The new amphipod species was found in four of the sampled cenotes and their respective submerged cave passages (Figure 1). These were Cenote Dzonbakal (Umán, 20.669819°N, 89.778869°W), Cenote Kanún (Homún, 20.745599°N, 89.244638°W), Cenote Xaan (Homún, 20.727571°N, 89.256834°W) and Cenote Kankirixché (Abalá, 20.37225°N, 89.632892°W). Amphipods were collected individually in 50 ml sample tubes during cave dives. Habitat data of each sample (depth, temperature, found in cavern or cave zone, caught in fresh water or saltwater) were recorded. Photos and videos of the observed crustaceans and their habitats were also taken. After the dives the collected individuals were placed into 96% ethanol. All specimens were collected under the permission of the Secretary of Environment and Natural Resources of United Mexican States (SEMARNAT/SPGA/DGVS/05263/14; SEMARNAT/SPGA/DGVS/02068/17). Type material is deposited in the National Crustacean Collection in the Institute of Biology of the National Autonomous University of México (UNAM), in the Yucatán Crustacea Collection at the Academic Multidisciplinary Unit of Teaching and Research of UNAM, and in the Collection of Crustaceans of the Hungarian Natural History Museum, as detailed in Table 1.

**Table 1.** Data of hadziid and hyalellid samples used for COI molecular studies. *Bahadzia jaraguensis* and *Hyalella azteca* sequences were downloaded from GenBank (http://www.ncbi.nlm.nih.gov/genbank).

Sample codes and collection codes	Species	Date of collection	Cenote, municipality, state	Collected by	Cited in	GenBank IDs
*Nr. 00046*HOLOTYPECNR 34392	*Mayaweckelia troglomorpha* sp. n.	14.05.2016	Dzonbakal, Umán, Yucatán	D. Angyal, R. Acosta, J. Baduy & S. Reyes	present study	MF589977
*Nr. 00113*ALLOTYPEHNHM Amphipoda -4094	*Mayaweckelia troglomorpha* sp. n.	11.06.2016	Cenote Kankirixché, Abalá, Yucatán	D. Angyal & E.M. Chávez Solís	present study	MF589981
*Nr. 00043*PARATYPECNR 34393	*Mayaweckelia troglomorpha* sp. n.	04.06.2016	Cenote Kanún, Homún, Yucatán	D. Angyal, R. Acosta, J. Baduy, B. Magaña & S. Reyes	present study	MF589976
*Nr. 00056*PARATYPEHNHM Amphipoda -4095	*Mayaweckelia troglomorpha* sp. n.	14.05.2016	Dzonbakal, Umán, Yucatán	D. Angyal, R. Acosta, J. Baduy & S. Reyes	present study	MF589978
*Nr. 00095*PARATYPEYUC-CC-255-11-003922	*Mayaweckelia troglomorpha* sp. n.	09.06.2016	Cenote Xaan, Homún, Yucatán	D. Angyal & E.M. Chávez Solís	present study	MF589979
*Nr. 00110*PARATYPEHNHM Amphipoda -4096	*Mayaweckelia troglomorpha* sp. n.	11.06.2016	Cenote Kankirixché, Abalá, Yucatán	D. Angyal & E.M. Chávez Solís	present study	MF589980
*Nr. 00042*YUC-CC-255-11-003923	*Mayaweckelia cenoticola* Holsinger, 1977	22.05.2016	Cenote Ayun-Nah, Cacalchén, Yucatán	D. Angyal & B. Magaña & E. Sosa Rodríguez	present study	MF589975
*Nr. 00108*YUC-CC-255-11-003924	*Tuluweckelia cernua* Holsinger, 1990	11.06.2016	Cenote Kankirixché, Abalá, Yucatán	D. Angyal & E.M. Chávez Solís	present study	MF589983
*Nr. 00101*YUC-CC-255-11-003925	*Tuluweckelia cernua* Holsinger, 1990	09.06.2016	Cenote Xaan, Homún, Yucatán	D. Angyal & E.M. Chávez Solís	present study	MF589982
*MX16.82*YUC-CC-255-11-003926	*Tuluweckelia cernua* Holsinger, 1990	11.12.2016	Cenote Concha (Sistema Sac Actun), Tulum, Quintana Roo	G. Balázs, B. Lerner, R. Mier & N. Kamarás	present study	MF589984
NC_019661.1	*Bahadzia jaraguensis* Jaume & Wagner, 1998	no data	Ovideo, S. Hispaniola (Dominican Republic), cave	no data	Bauzá-Ribot et al. 2012	NC019661.1
HM_138032.1	*Hyalella azteca* (Saussure, 1858)	no data	Canada, within the frame of Canadian Aquatic Biomonitoring Network program	no data	Baird et al. 2011	HM138032.1

### Morphological studies

Selected specimens of the presumably new species and other Yucatán hadziids (*M. cenoticola* and *T. cernua*) were dissected on slides and were studied under compound (light) microscope. At first, they were heated in 10% KOH solution, rinsed with HCl and washed in distilled water. Cleared exoskeletons were stained with chlorazol black, partly dissected in glycerol, and mounted on slides in glycerol gelatine medium under a stereomicroscope (Fišer et al. 2009, Angyal et al. 2015). Slides were examined using a Leica DM 1000 light microscope. Drawings were made using a drawing tube mounted on the light microscope and were digitally edited afterwards. Scanning electron micrographs were made with a HITACHI S-2600 N scanning electron microscope. The studied specimen was placed in absolute alcohol for one day, then was dissected and dried out in air. Dry samples were stuck onto holders and were sputter-coated with gold-palladium. Micrographs were digitally edited. The terminologies ’slender seta’ and ’robust seta’ were based on Watling’s (1989) classification system for crustacean setae. The terms ’notched spine teeth’ and ’unnotched spine teeth’ are based on the descriptions of Yucatán subterranean hadziids (Holsinger 1977, 1990), and refer to strong, thick spine-like features, typically on the palm of gnathopod I and II propodus.

### Molecular studies

DNA extraction of six individuals of *Mayaweckelia troglomorpha* sp. n., one *M. cenoticola* specimen and three *T. cernua* individuals (two from Yucatán state and one from Quintana Roo state, see Table 1 for sample data) was performed using QIAamp DNA Microkit (Qiagen), following the manufacturer’s instructions. Only a few pereopods were used for DNA isolation of each animal. For PCR amplification of mitochondrial cytochrome c oxidase subunit I (COI) we used the primer pair LCO 1490 and HCO 2198 (Folmer et al. 1994). PCR reactions (25 µl) were obtained by mixing 13.85 µl mQ water, 2.5 µl 10X PCR buffer, 2.5 µl dNTP mix (2mM), 1.5 µl of each primers (5µM), 0.15 µl Fermentas Dream Taq (5U/ µl) and 3 µl DNA extract. PCR temperature conditions were as follows: initial denaturation for 3 min at 94 °C, denaturation for 45 sec at 94 °C, hybridization for 45 sec at 48 °C, and polymerization for 1 min at 72 °C. After thirty cycles a final extension for 3 min at 72 °C was added. PCR products were cleaned using Exo SAP-IT Express PCR Product Cleanup (Affymetrix) according to manufacturer’s instructions. The fragments were sequenced in both directions using PCR amplification primers with an ABI 3130 sequencer. 638 bp COI barcode sequences have been uploaded to NCBI GenBank database. Accession numbers are MF589975–MF589984 (see Table 1).

### Sequence analyses

In order to evaluate phylogenetic relationships and genetic distances of the newly collected hadziids (*Mayaweckelia* spp. and *T. cernua*) with other hadziid and hyalellid species with publicly available sequences, a dataset of COI sequences was compiled (Table 1). The widespread and abundant north and central American *Hyalella azteca* (Hyalellidae) was included in the dataset as outgroup taxon.

DNA sequences were edited using BioEdit 7.1.11 (Hall 1999) and aligned with ClustalW multiple sequence alignment program (Thompson et al. 1994). Nucleotide substitution model selection carried out with MEGA V 6.0 (Tamura et al. 2013) using the Akaike information criterion (AIC) (Akaike 1973) revealed that the best fitting model for COI is GTR+G+I. Bayesian inference was carried out on phylogeny.fr (Dereeper et al. 2008) using Metropolis coupled Markov chain Monte Carlo simulations for 100.000 generations, sampling a tree in every 10 generations. The first 1000 trees were discarded as burn-in. FigTree 1.4.0 (Rambaut 2012) was used for visualisation. Pairwise genetic distances were calculated in MEGA V 6.0 using p-distance (Nei and Kumar 2000).

## Results

### Taxonomy

#### Order Amphipoda Latreille, 1816

##### Suborder Senticaudata Lowry & Myers, 2013

###### Family Hadziidae S. Karaman, 1943

####### Genus *Mayaweckelia* Holsinger, 1977

######## Mayaweckelia troglomorpha

Taxon classificationAnimaliaAmphipodaHadziidae

Angyal sp. n.

http://zoobank.org/32D988B9-58D3-4224-9A21-53B9C2BFB8F5

Figs 2 , 3 , 4 , 5 , 6 , 7 , 8 , 9

######### Material examined.

*Holotype* ♂, 10 mm, Nr. 00046, 14 May 2016, Dzonbakal, 20.669819°N, 89.778869°W, San Antonio Mulix, Umán, Yucatán state, México, collected by D. Angyal, R. Acosta, J. Baduy & S. Reyes in cave part, 26.7 m depth in fresh water; dissected and mounted on slide. Collection ID: CNR 34392 (UNAM, Institute of Biology, National Crustacean Collection, México City.)

*Allotype* ♀, 10 mm, Nr. 00113, 11 June 2016, Cenote Kankirixché, 20.37225°N, 89.632892°W, Mucuyché, Abalá, Yucatán state, México, collected by D. Angyal & E.M. Chávez Solís in cavern part, 20.4 m depth in fresh water; dissected and mounted on slide. Collection ID: HNHM Amphipoda -4094 (Hungarian Natural History Museum, Collection of Crustaceans, Budapest).

*Paratypes* ♀, 7 mm, Nr. 00056, 14 May 2016, Dzonbakal, 20.669819°N, 89.778869°W, San Antonio Mulix, Umán, Yucatán state, México, collected by D. Angyal, R. Acosta, J. Baduy & S. Reyes in cave part, 26.3 m depth in fresh water; sputter-coated by gold-palladium. Collection ID: HNHM Amphipoda -4095 (Hungarian Natural History Museum, Collection of Crustaceans, Budapest).

♂, 8 mm, Nr. 00043, 4 June 2016, Cenote Kanún, 20.745599°N, 89.244638°W, Homún, Homún, Yucatán state, México, collected by D. Angyal, R. Acosta, J. Baduy, B. Magaña & S. Reyes in cave part, 24.3 m depth in fresh water; not dissected. Collection ID: CNR 34393 (UNAM, Institute of Biology, National Crustacean Collection, México City).

Juvenile, 3 mm, Nr. 00095, 9 June 2016, Cenote Xaan, 20.727571°N, 89.256834°W, Homún, Homún, Yucatán state, México, collected by D. Angyal & E.M. Chávez Solís in cave part, 25.4 m depth in fresh water; not dissected. Collection ID: YUC-CC-255-11-003922 (UNAM, Academic Multidisciplinary Unit of Teaching and Research, Yucatán Crustacea Collection, Sisal).

Juvenile, 5 mm, Nr. 00110, 11 June 2016, Cenote Kankirixché, 20.37225°N, 89.632892°W, Mucuyché, Abalá, Yucatán state, México, collected by D. Angyal & E.M. Chávez Solís in cave part, 33.3 m depth in fresh water; dissected and mounted on slide. Collection ID: HNHM Amphipoda -4096 (Hungarian Natural History Museum, Collection of Crustaceans, Budapest).

######### Diagnosis.

Medium-sized, eyeless hadziid with conspicuous troglomorphic traits. The first antenna almost twice as long as body and three times as long as the second antenna; gnathopod I propodus palm armed with distally notched spine teeth, carpus more than 1.5 times as long as corresponding propodus, merus as broad as but shorter than carpus, ventrally produced lobe with three long sensory setae; gnathopod II propodus twice as long as propodus I, palm armed with unnotched spine teeth, carpus slightly shorter than propodus on males. Dactylus, propodus. and carpus of pereopods VI-VII extremely long; therefore, pereopods VI and VII are 1.3 times as long as body length; epimeral plates I-III ventro-posterior corner tiny but distinct, ventral margin without robust setae, posterior margins concave; surfaces of uropods I-III pubescent; telson lobes each possess five-six robust setae and one-three slender setae on outer margin and six-seven robust setae on inner margin. Largest males and females both measured 10 mm.

######### Description.

**Figure 2.**
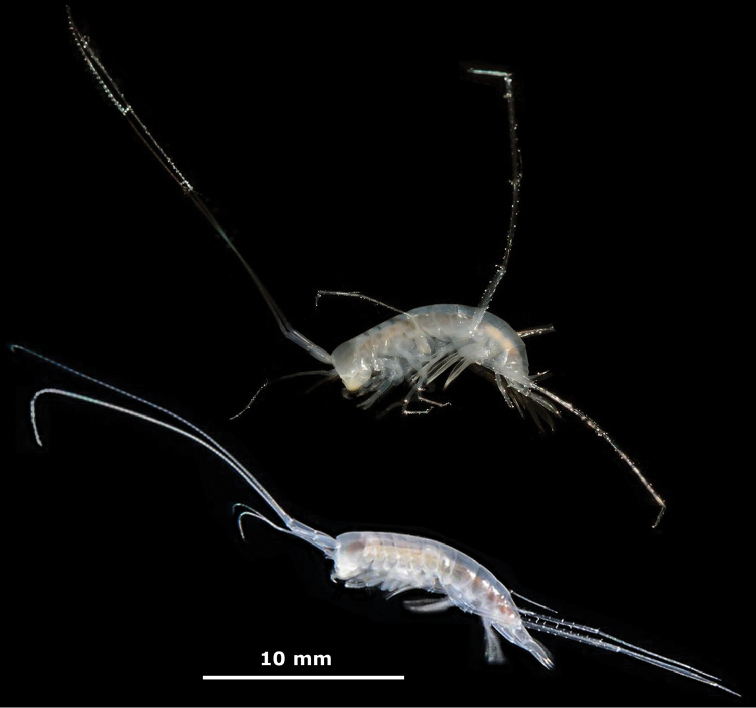
*M. troglomorpha* sp. n., living specimens. Above: allotype ♀ collected in Cenote Kankirixché; below: individual photographed in its natural habitat during research dive in Cenote Kanún (not collected).

**Figure 3.**
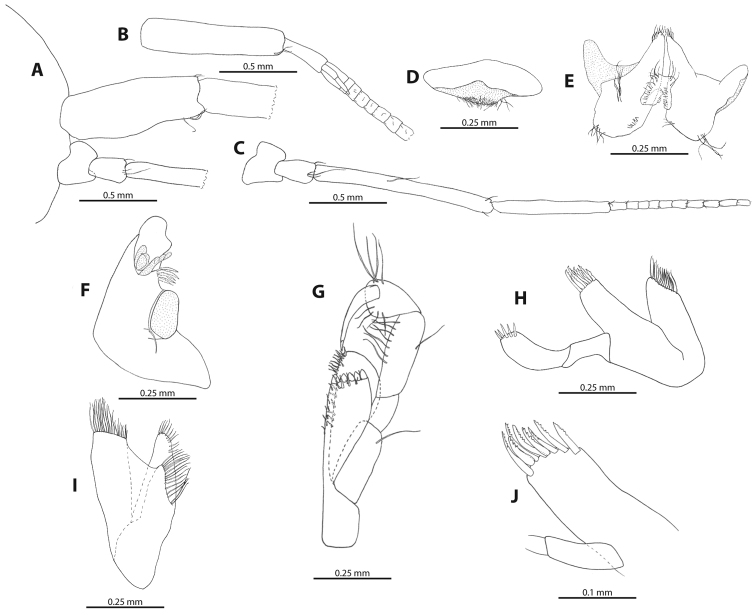
*M. troglomorpha* sp. n. **A** detail of head and antenna I and II (♀ allotype) **B** detail of antenna I with accessory flagellum (♀ allotype) **C** antenna II peduncle articles and proximal part of flagellum (♀ allotype) **D** upper lip (♂ holotype) **E** lower lip (♂ holotype) **F** left mandible (♀ allotype) **G** maxilliped (8 mm ♂) **H** maxilla 1 (♀ allotype) **I** maxilla II (♀ allotype) **J** maxilla I outer plate (♂ holotype).

(10 mm ♂, 8 mm ♂, 8 mm ♀, 7 mm ♀, 5 mm juvenile, 3 mm juvenile.) Antenna 1 (Figures 2, 3) 1.75 times as long as body; three times long as antenna II; primary flagellum with more than 60 articles; aesthetacs were not visible as distal half of the flagellum was missing from each animal (examination of the antennae was possible only using the photos of the living specimens); accessory flagellum with three articles. Antenna II (Figures 2, 3): flagellum with more than 20 articles. Mandibles (Figure 3) subequal; both molar with seta; setal row with four or five serrated setae; palp lacking. Maxilla I (Figure 3): inner plate with approximately 15 apical setae; outer plate with eight apical, pluri-toothed robust setae; second palpal article with five apical robust setae. Maxilla II (Figure 3): inner plate with 15–23 obliquely placed setae on inner margin. Maxilliped (Figure 3): inner plate with four or five cone shaped, thick robust setae and several coarse setae apically; outer plate with stiff setae apically. Lower lip (Figure 3): outer lobes narrowly rounded; lateral process prominent; inner lobes rather small.

**Figure 4.**
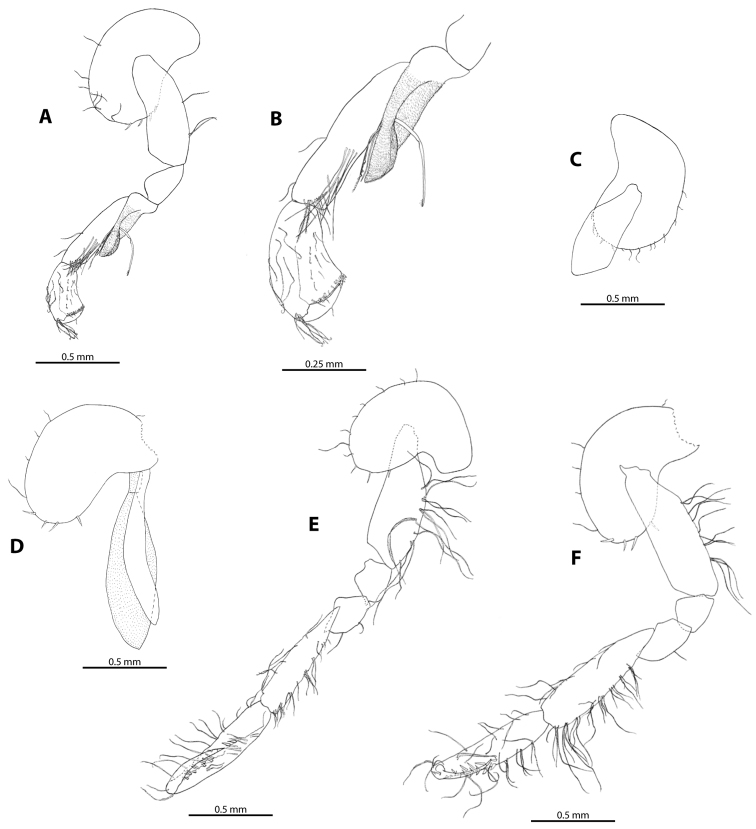
*M. troglomorpha* sp. n. **A** gnathopod I (♀ allotype) **B** gnathopod I propodus, carpus and merus (♀ allotype) **C** gnathopod I coxa (♂ holotype) **D** oostegite and gill on gnathopod II (♀ allotype) **E** gnathopod II (♂ holotype) **F** gnathopod II (♀ allotype).

**Figure 5.**
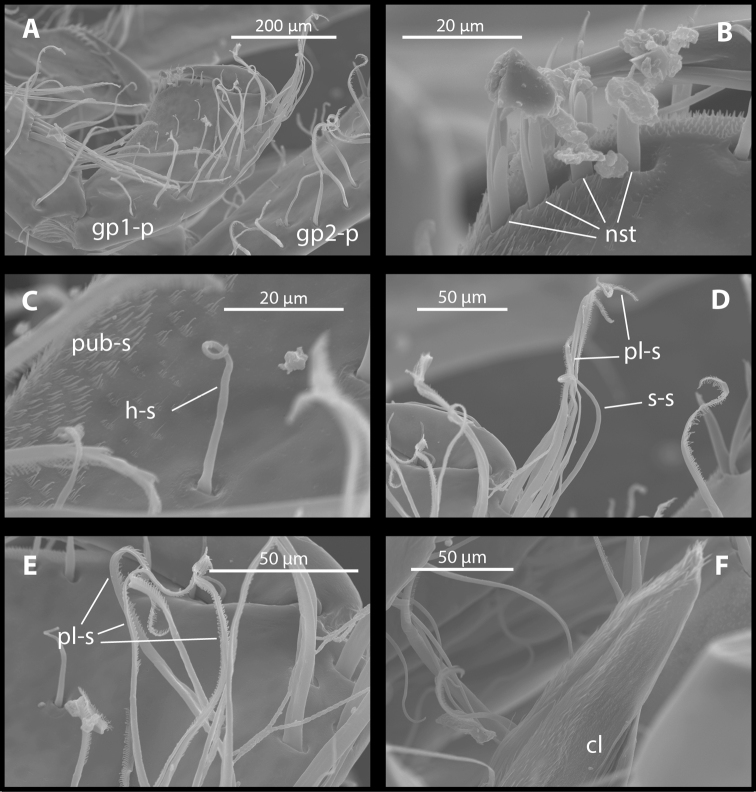
*M. troglomorpha* sp. n., (7 mm ♀), scanning electron micrographs. **A** gnathopod I propodus; **B**, gnathopod I palmar corner **C** gnathopod I propodus posteromedial part **D** gnathopod I propodus anterodistal seta group **E** gnathopod I propodus anterior margin seta group **F** ventrally produced conspicous lobe on gnathopod I merus. Abbreviations: gp1-p = gnathopod I propodus, gp2-p = gnathopod II propodus (**A**); nst = notched spine teeth (**B**); pub-s = pubescent setae, h-s = helical medial seta (**C**); pl-s = plumose seta, s-s = simple seta (**D**); pl-s = plumose seta (**E**); cl = conspicous lobe (**F**).

Gnathopod I (Figure 4, 5): dactyl thick, single seta present on anterior margin, inner margin without seta, unguis (nail) length 35% of total dactylus length. Propodus small, longer than broad; palm short, slightly convex, in palmar corner double row of four-five distally notched spine teeth always present, additional notched spine teeth and spine-like setae sometimes present on palm, close to the base of dactylus; posterior margin of propodus slightly concave, surface near margin covered with pubescent setae; anterior margin with five-seven rows of long, plumose setae (sometimes singly inserted); antero-distal group with six-eight long plumose and simple setae; four singly inserted helical medial setae always present, sometimes additional singly or doubly inserted medial setae present. Carpus narrow, 1.5–1.7 times longer than propodus. Merus: as broad as but shorter than carpus, ventrally produced into pubescence, conspicuous lobe with three long sensory setae. Sensory papillae visible on one of the setae. Coxal plate I large, deep, longer than broad, broadly rounded ventrally, margin with three-four robust setae and seven-eight slender setae on females and six robust setae and three-eight slender setae on males.

**Figure 6.**
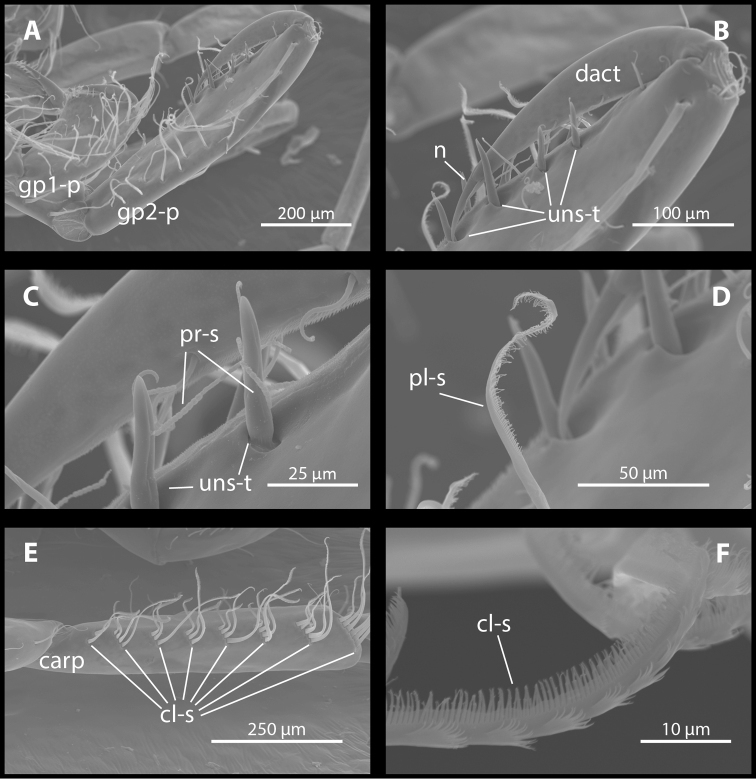
*M. troglomorpha* sp. n., (7 mm ♀), scanning electron micrographs. **A** gnathopod I and II propodus **B** gnathopod II propodus dactylus and palm **C** gnathopod II propodus palm **D** gnathopod II propodus posterior margin **E** gnathopod II carpus **F** comb-like plumose seta on gnathopod II carpus. Abbreviations: gp1-p = gnathopod I propodus, gp2-p = gnathopod II propodus (**A**); dact = dactylus, n = nail, unst-t = unnotched spine teeth (**B**); pr-s = pearl row-like seta, unst-t = unnotched spine teeth (**C**); pl-s = plumose seta (**D**); carp = carpus, cl-s = comb-like plumose setae (**E**); cl-s = comb-like plumose seta (**F**).

Gnathopod II (Figure 4, 6): dactylus thick, along anterior margin (close to antero-distal corner) a single seta present, inner margin with three-five setae. Propodus twice as long as gnathopod I propodus, narrow, subrectangular; palm length is more than 50% of propodus length on males and less than 50% of propodus length on females; palm armed with double row of five-six unnotched spine teeth which are sometimes accompanied by long, pearl row-like setae; surface near margin covered with pubescent setae; helical seta sometimes present below (proximal to) spine teeth; posterior margin with four-five, anterior margin with six-nine sets of plumose setae; three-four medial plumose setae. Carpus slightly shorter than propodus on males and slightly longer than propodus on females, armed with seven-eight rows of comb-like plumose setae on posterior margin. Merus as broad as but more than two times shorter than carpus; not produced ventrally into conspicuous lobe. Coxal plate II kidney-shaped, margin with three-four robust setae and eight slender setae.

**Figure 7.**
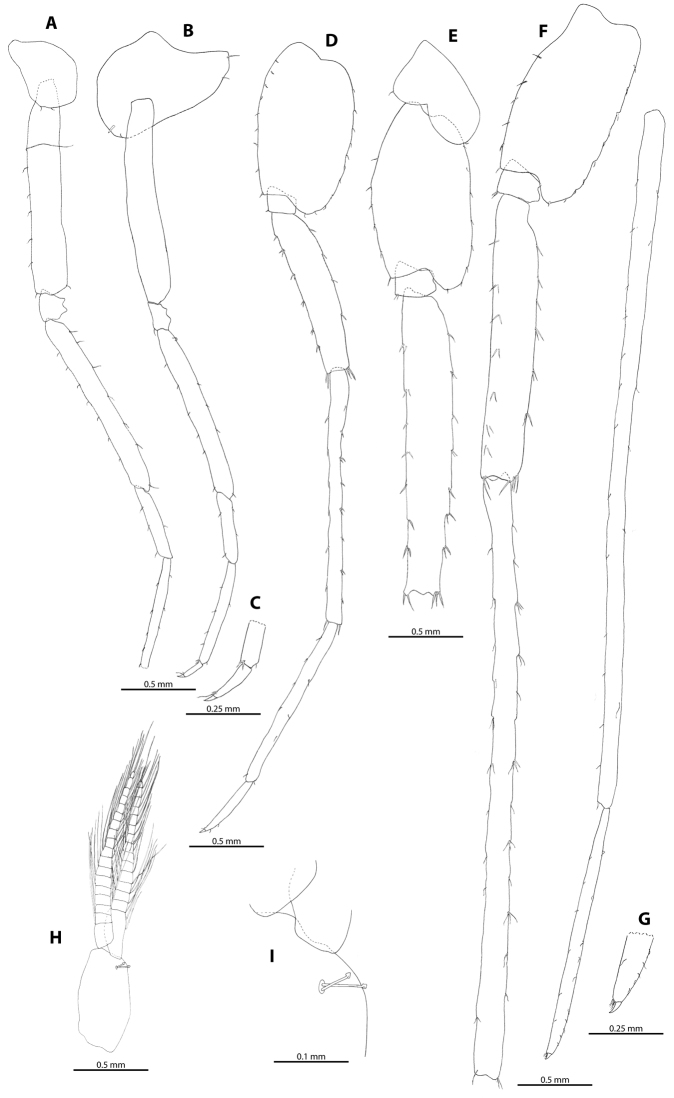
*M. troglomorpha* sp. n., ♂ holotype. **A** pereopod III (dactylus was missing) **B** pereopod IV **C** pereopod IV dactylus **D** pereopod V **E** pereopod VI coxa, basis, ischium and merus **F** pereopod VII **G** distal part of pereopod VII with detail of unguis **H** pleopod III **I** retinacle on pleopod II.

Coxal plate III (Figure 7) rather small and shallow, margin with two fine setae. Coxal plate IV (Figure 7) more than twice as broad as coxal plate III; posterior margin concave, ventral margin with four short stiff setae; dactylus length 28% of propodus; single robust seta and one long slender seta at the base of the unguis. Pereopod V (Figure 7) basis 1.7 times longer than broad, margins convex; pereopods VI (Figure 7) and VII (Figure 7) bases twice as long as broad, margins of pereopod VI slightly convex, posterior margin of pereopod VII straight; dactylus of pereopod V 40% length of corresponding propodus; outer margin with two slender setae; pereopod VI and VII extremely long (especially dactylus, propodus and carpus), 1.3 times as long as body; pereopod VI slightly longer than pereopod VII; pereopod VII dactylus with some short, slender setae at the base of the unguis. Coxal gills (Figure 4) large, almond shaped, pedicellate, present on pereon segments II-VI; oostegites (Figure 4) long, slender. Pleopods (Figs 7, 9) I-III with two-hooked retinaculae.

**Figure 8.**
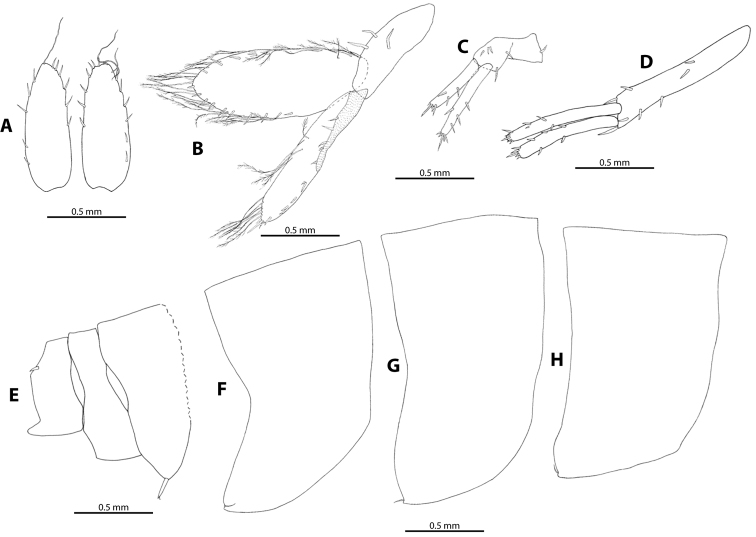
*M. troglomorpha* sp. n. **A** telson (♀ allotype) **B** uropod III (♀ allotype) **C** uropod II (♂ holotype) **D** uropod I (♂ holotype) **E** urosomites (♀ paratype, 7 mm) **F** pleonite III (♀ allotype) **G** pleonite II (♀ allotype) **H** pleonite I (♀ allotype).

Epimeral plates I-III (Figure 8) ventro-posterior corner tiny but distinct with one slender seta at the corner; ventral margin without robust setae; posterior margin slightly concave on epimeral plate I, concave on epimeral plate II and strongly concave on epimeral plate III. Urosomites (Figure 8): urosomite I with one strong robust seta at the base of uropod I; urosomite II without robust and slender setae; urosomite III with one robust seta mid-dorsally.

**Figure 9.**
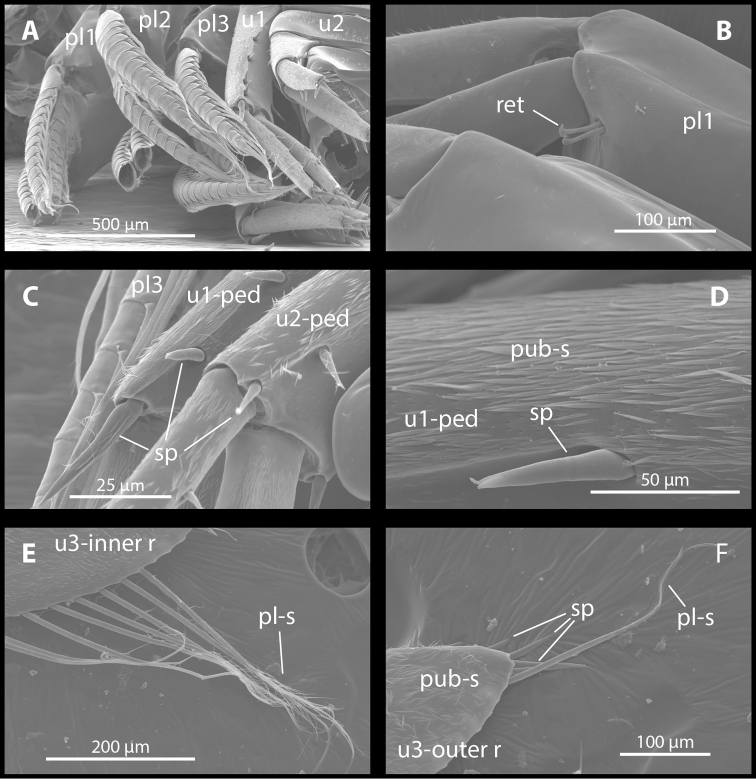
*M. troglomorpha* sp. n., (7 mm ♀), scanning electron micrographs. **A** pleopods I-III and uropods I and II **B** retinacle on pleopod I; **C**, spine-like robust setae on uropod I and II **D** detail of uropod I peduncle article **E** setae on uropod III inner ramus **F** apical region of uropod III outer ramus. Abbreviations: pl1 = pleopod I, pl2 = pleopod II, pl3 = pleopod III, u1 = uropod I, u2 = uropod II (**A**); ret = retinacle, pl 1 = pleopod I (**B**); pl3 = pleopod III, u1-ped = peduncle article of uropod I, u2-ped = peduncle article of uropod II, sp = spine-like robust setae (**C**); pub-s = pubescence setae, u1-ped = uropod I peduncle article, sp = spine-like robust seta (**D**); pa-s = pappose seta, u3-inner r = uropod III inner ramus (**E**); pl-s = plumose seta, sp = spine-like robust seta, pub-s = pubescent setae, u3-outer r = uropod III outer ramus (**F**).

Uropod I (Figures 8, 9) surface of rami and peduncle covered with pubescent setae; outer ramus 10–15% longer than inner ramus, outer ramus 20–33% shorter than peduncle; peduncle with five-eight spine-like robust setae; outer ramus with four-six robust setae (plus five apical robust setae), inner ramus with five-six robust setae (plus five apical robust setae). Uropod II (Figures 8, 9) surface of rami and peduncle covered with pubescent setae; outer ramus 13–22% longer than inner ramus on the studied males and 25–35% longer than inner ramus on females; outer ramus 5–15% longer than peduncle; peduncle with six spine-like robust setae; inner ramus with three-four robust setae (plus five apical robust setae); outer ramus with four-five robust setae (plus five apical robust setae). Uropod III (Figures 8, 9) surface of rami and distal end of peduncle covered with pubescent setae; uropod III 20% as long as body; inner ramus slightly longer than outer ramus, margins with long, singly-inserted plumose and pappose setae and some short robust setae, apex with two short spine-like robust setae; outer ramus with long, plumose setae on inner margin and short robust setae on outer margin toward distal end, apex with three robust setae and sometimes additional with long spine-like seta; peduncle with two-four robust setae.

Telson (Figure 8) cleft to base, each half 2.65 times longer than broad; lobes each with five-six robust setae and one-three slender setae on outer margin and six-seven robust setae on inner margin.

######### Variability.

Sexes very similar in size and appearance, except a few traits. Propodus I more oblong in males than in females. Coxal plate I margin with six robust setae and three-eight slender setae in males, and with three or four robust setae and seven-eight slender setae in females. Palm length of gnathopod II is more than 50 % of propodus length on males and less than 50 % of propodus length on females. Carpus of gnathopod II slightly shorter than propodus on males and slightly longer than propodus on females. Sexually mature females have long and narrow oostegites. Left and right side gnathopod I and II are unequal in length in both sexes. Outer ramus of uropod II 13–22 % longer than inner ramus on males and 25–35 % longer than inner ramus on females, however this character should be further analysed on an elevated number of individuals of both sexes.

######### Etymology.

The name *troglomorpha* refers to the highly adaptive troglomorphic features of the new species, particularly the elongation of appendages, the increased number of sensory setae and papillae, and general appearance of fragility. Gender feminine.

######### Distribution and remarks on ecology.

The new species is known from four cenotes in the state of Yucatán, covering a distribution distance of 52 km (distance between the farthest cenotes Dzonbakal and Xaan). All the individuals were found in fresh water habitat, in most cases far from the cenote entrances, deeper in the associated cave passages, where sunlight does not penetrate. Water temperature was between 26 and 27 °C. Specimens were collected between 20 and 33 meters depth; in cenote Kankirixché some individuals were observed below 45 meters depth. The new species was represented in all four localities with low abundance, though it proved to be more common and more abundant than *M. cenoticola*, of which a single specimen was found in only one (Ayun-Nah) of the 14 visited cenotes, during an underwater waste collecting activity, hidden in a plastic soft drink bottle. In the type locality and in cenotes Xaan and Kankirixché the new species co-occurred with the hadziid amphipod *Tuluweckelia cernua*. Other co-occurring stygobiont macro-crustaceans (in the four cenotes) were the mysid *Antromysis cenotensis* Creaser, 1936, the stygiomysid Stygiomysis cf. holthuisi (Gordon, 1958), the isopods *Creaseriella anops* (Creaser, 1936) and *Yucatalana robustispina* Botosaneanu & Iliffe, 1999, and the decapods *Typhlatya mitchelli* Hobbs & Hobbs, 1976, *Typhlatya pearsei* Creaser, 1936, and *Creaseria morleyi* (Creaser, 1936).

######### Remarks and affinities to related species and genera.

Holsinger (1990) noted that his original description of *M. yucatanensis* (Holsinger 1977) ‘was based on what appear to be submature specimens, therefore raising the strong possibility that the differences noted between the two species of *Mayaweckelia* are due primarily to age’, and the two species probably should be synonymized. However, the synonymisation has not been published until now. *Mayaweckelia troglomorpha* sp. n. differs from *M. yucatanensis* by i) three times larger body size; ii) significantly increased number of flagellum articles in both antennae (three times more articles on primary flagellum); iii) gnathopod I carpus 1.5–1.7 times longer than propodus (vs. same length); iv) proportionally longer and differently ornamented propodus of gnathopod II (palm armed with unnotched spine teeth and pearl row-like setae); v) pereopods VI and VII 130 % of body length (vs. 60 % of body length) vi) more distinct ventro-posterior corner of epimeral plates. *M. troglomorpha* sp. n. differs from *M. cenoticola* by i) its two times larger body size; ii) elevated number of flagellum articles in both antennae; iii); less narrow and differently ornamented propodus of gnathopod I (palm armed with distally notched spine teeth); iv) gnathopod I carpus 1.5–1.7 times longer than propodus (vs. 0.7 times longer); v) longer and differently ornamented propodus of gnathopod II; vi) pereopods VI and VII 130 % of body length (vs. approximately 60 % of body length); and vii) more distinct ventro-posterior corner of epimeral plates. Scanning electron microscopy has revealed that uropods I–III are covered with pubescent setae (not mentioned in the description of *M. yucatanensis* and *M. cenoticola*), this character should also be checked on these species using SEM studies, as this trait is not visible using light microscopy.

The new species corresponds with the diagnostic characters of the genus *Mayaweckelia*. It differs from the related *Tuluweckelia* in the following traits: i) anterior body region does not bend markedly downward; ii) maxilla I outer lobe with seven-nine setae; iii); gnathopod II sexually dimorphic; iv) epimeral plates ventro-posterior corners less produced. *Mayaweckelia* differs from *Bahadzia* by i) the absense of palp from both mandibles; and ii) outer ramus of uropod III with one article (Holsinger and Yager 1985, Holsinger 1992). *Mexiweckelia* Holsinger & Minckley, 1971 and *Paramexiweckelia* Holsinger, 1982 are subterranean genera of the ‘weckeliid’ group known from north of México (e.g., Holsinger and Minckley 1971, Holsinger 1982). *Mayaweckelia* differs from them in some important ways: i) accessory flagellum of first antenna three-articulated (vs. single or vestigial); ii) presence of robust setae on inner margin of maxilliped outer lobe; iii) presence of large, ventrally produced lobe on gnathopod I merus; iii) sexually dimorphic gnathopod II (of *Paramexiweckelia* is not dimorphic); iv) pereopod VI little longer than pereopod VII; and v) completely separated telson halves (vs. deeply incised but fused in the other two genera).

### Mitochondrial gene sequences

**Figure 10.**
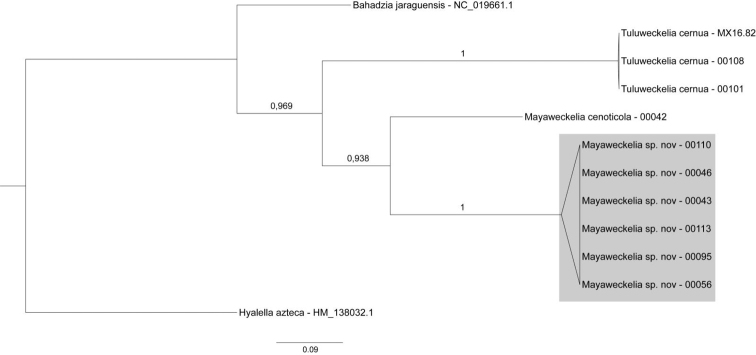
Bayesian phylogenetic tree of COI sequences based on the collected *Mayaweckelia* and *Tuluweckelia* samples and publicly available hadziid and hyalellid sequences. *Hyalella azteca* was included as outgroup taxon. Posterior probability values are indicated. *Bahadzia jaraguensis* and *H. azteca* sequences are after Bauza-Ribot et al. (2012) and Baird et al. (2011), respectively.

In accordance with the morphological data, the Bayesian analysis of COI sequences showed that the closest relative of the herein described new species is *M. cenoticola*, forming a monophyletic group referring to the genus *Mayaweckelia* (Figure 10). Comparing uncorrected p-distances (Table 2), the distance between *M. cenoticola* and the new species is 22 % (p = 0.221–0.224). Five individuals of *M. troglomorpha*, sp. n. show rather low intraspecific variance (p = 0.002–0.009). Among these, all substitutions proved to be synonymous (same sense), occurring in the third codon positions. However, individual ‘00110’ of the new species differs in 2 % (p = 0.016–0.022) from the other five specimens, and contains a nonsynonymous substitution. COI sequences of the three *Tuluweckelia cernua* individuals, including the one which was collected in Quintana Roo state, belonged to the same haplotype. Though, mitochondrial gene sequence of only a single species of the genus *Bahadzia* was available, the constructed phylogenetic tree is in accordance with the taxonomical ranks, *Tuluweckelia* being the sister group of *Mayaweckelia*. It is worth mentioning that the uncorrected p-distance value between the two *Mayaweckelia* species is almost as high (22 %) as the distance between the three genera (24–30 %).

**Table 2.** Uncorrected p-distances between the studied hadziid species.

		**1**	**2**	**3**	**4**	**5**	**6**	**7**	**8**	**9**	**10**
**1**	*Mayaweckelia cenoticola* -00042										
**2**	*M. troglomorpha* sp. n. -00110	0.221									
**3**	*M. troglomorpha* sp. n. -00046	0.224	0.022								
**4**	*M. troglomorpha* sp. n. -00113	0.224	0.016	0.008							
**5**	*M. troglomorpha* sp. n. -00095	0.223	0.017	0.009	0.002						
**6**	*M. troglomorpha* sp. n. -00043	0.224	0.022	0.003	0.008	0,009					
**7**	*M. troglomorpha* sp. n. -00056	0.224	0.017	0.009	0.002	0,003	0.009				
**8**	*Tuluweckelia cernua* -MX16.820	0.265	0.288	0.296	0.296	0,298	0.296	0.295			
**9**	*Tuluweckelia cernua* -00108	0.265	0.288	0.296	0.296	0,298	0.296	0.295	0.000		
**10**	*Tuluweckelia cernua* -00101	0.265	0.288	0.296	0.296	0,298	0.296	0.295	0.000	0.000	
**11**	*Bahadzia jaraguensis* -NC 019661.1	0.251	0.248	0.243	0.246	0,245	0.241	0.246	0.277	0.277	0.277

## Discussion

The ‘weckeliid’ group of Hadziidae is composed of mostly monotypic, predominantly subterranean freshwater genera with a significant concentration of species in the old Tethyan remnants of the greater Caribbean and Gulf of México regions (e.g., Holsinger and Longley 1980, Barnard and Barnard 1983, Holsinger 1986, Holsinger and Ruffo 2002). Their evolution into freshwater stygobionts is explained by ‘stranding’ in newly developing hypogean freshwater habitats following marine regressions (e.g., Holsinger 1977, Stock 1980, Holsinger and Longley 1980, Holsinger 1986, 1992, 1994, Holsinger and Ruffo 2002). Holsinger (1986) stated that this group is primarily distinguished morphologically from other members of the family Hadziidae by the apomorphic character state of the third uropod. In the weckeliids, the rami are typically subequal in length and the outer ramus lacks a second article. A further important weckelioid character is the lack of mandibular palp (Stock 1985). Primarily because of the former two characters, *Mayaweckelia* and *Tuluweckelia* were previously considered to be members of the weckeliid group. However, as Holsinger (1990) pointed out, unlike all other genera previously classified to the weckeliids, the two Yucatán genera lack basofacial robust setae on the first uropod. Later on, Holsinger and Ruffo (2002) recommended the two genera to be assigned to separate groups, as they apparently belong to other lineages within the family.

Cladistic analysis performed including *Bahadzia*, the third stygobiont hadziid genus known from the peninsula and another 13 further hadziid genera suggested that *Mayaweckelia* and *Tuluweckelia* are sister genera to *Bahadzia* and may even be derived from a *Bahadzia*-like ancestor (Holsinger 1992, Sawicki and Holsinger 2004). Our mitochondrial sequence analysis supports this idea, though, it would be necessary to collect individuals of both Yucatán *Bahadzia* species (*B. bozanici* and *B. setodactylus*) to further solve this question.

*Mayaweckelia cenoticola* was previously recorded in 13 caves and cenotes and, except for two or three, they were all taken from fresh water habitats (Holsinger 1977, Reddell 1981, Holsinger 1990, Rocha et al. 1998, Alvarez and Iliffe 2008, Alvarez et al. 2015). Individuals of the new *Mayaweckelia* species were also found in freshwater in all cases, as well as the single specimen of newly collected *M. cenoticola*.

Intergeneric sympatry of subterranean Hadziidae, which is quite rare, can be explained by secondary contact (Bouin and Messouli 1988). This study revealed that the monotypic genus *Tuluweckelia*, which was previously known mostly from saltwater habitats of anchialine cenotes near the northeastern coastline of the peninsula (Holsinger 1990, Rocha et al. 1998, Alvarez and Iliffe 2008, Alvarez et al. 2015), exists in freshwater cenotes and submerged cave passages far from the coastline as well. *Tuluweckelia cernua* proved to be a relatively common species of the visited localities, as we found small populations in almost half of the sampled localities. Referring to the species’ geographic distribution and ecology, Holsinger (1990) considered that the origin of *Tuluweckelia* from hypothetical marine ancestors is more recent than that of *Mayaweckelia* and ’may be related to the recession of a high sea stand during the Pleistocene’. Interestingly, despite the approximately 200 km distance between the most distant localities, only one COI haplotype occured within the individuals collected in Yucatán state and Quintana Roo state (see sample data in Table 1). Botello and Alvarez (2010) pointed out that in case of the Yucatán cave shrimp *Creaseria morleyi*, genetic variation is a relict of an ancient marked genetic structure reduced by changes in sea level that resulted in a series of bottlenecks. A support to *Tuluweckelia*’s more recent marine originated subterranean colonisation hypothesis (Holsinger 1990) can be that unlike *Mayaweckelia*, during our thorough samplings in 14 cenotes, we have not discovered additional species of the genus *Tuluweckelia*, other than *T. cernua*. To study the origin of the peninsula’s stygobiont hadziid fauna and to calibrate divergence times, an extended phylogenetic study would be needed, involving a series of species from different habitat types, using both mitochondrial and nuclear markers.

It is remarkable that in spite of the low intraspecific variability recorded of *M. troglomorpha* sp. n., COI sequence of one individual differed significantly from all the rest. This individual was found in Cenote Kankirixché, which is characteristically holds the most diverse subterranean crustacean fauna among the studied cenotes. In the same site, another individual of the new species was also collected, which shared the same haplogroup with the rest of the specimens from other cenotes. These two individuals were found in two distinct parts of the system: the former far from the entrance, below 30 meters depth in a descending cave passage, while the latter closer to the entrance, in the cavern part. To study the possibility of cryptic speciation, further molecular studies of additional samples from Kankirixché could lead to interesting results.

Obtaining individuals for morphological and molecular genetic analyses from the type locality of *M. yucatanensis* (Grutas de Xtacumbilxunam, Campeche state) could aid in a comparison and validation of the species. To gain a better knowledge on the distribution range of the previously known and the newly described *Mayaweckelia* species and to contribute to their conservation planning, it would be important to explore additional cenotes and other subterranean ecosystems in Yucatán state and in the rest of the peninsula. Local regulations that target the protection of the species’ habitats are necessary.

## Conclusions

To date, only a small proportion of the cenotes and other aquatic hypogean ecosystems have been studied in Yucatán state in zoological aspect. Our expedition has led to the discovery of a new species of subterranean hadziids, which confirms that exploration and further studies of the region’s groundwater Crustacea diversity is necessary. Description of the new species was completed with comparative scanning electron microscopy, which was used for first time on *Mayaweckelia*. It proved to be a rather useful method for discovering, analysing, and illustrating barely visible diagnostic characters. As contributions to the future molecular genetic studies on Yucatán subterranean hadziids, COI sequences as barcodes of *M. troglomorpha* sp. n., *M. cenoticola*, and *T. cernua* are now publicly available in GenBank. The phylogenetic studies have shown that based on the available sequences, the closest relative of the new species is *M. cenoticola*. In accordance with the previous cladistic studies, *Mayaweckelia* and *Tuluweckelia* prove to be sister genera, closely related to *Bahadzia*, the third Yucatán subterranean Hadziidae genus. This knowledge may contribute to the species’ future conservation planning.

## Supplementary Material

XML Treatment for Mayaweckelia troglomorpha
